# Efficacy and Safety of Photon Induced Photoacoustic Streaming for Removal of Calcium Hydroxide in Endodontic Treatment

**DOI:** 10.1155/2018/2845705

**Published:** 2018-04-23

**Authors:** Markus Laky, Melanie Volmer, Muazzez Arslan, Hermann Agis, Andreas Moritz, Barbara Cvikl

**Affiliations:** ^1^Division of Conservative Dentistry and Periodontology, School of Dentistry, Medical University of Vienna, Vienna, Austria; ^2^Division of Dental Student Training and Patient Care, School of Dentistry, Medical University of Vienna, Vienna, Austria; ^3^Department of Preventive, Restorative and Pediatric Dentistry, School of Dentistry, University of Bern, Bern, Switzerland

## Abstract

Calcium hydroxide removal from the root canal by photon induced photoacoustic streaming (PIPS) compared to needle irrigation and irrigation using sonic activation was investigated. Additionally, safety issues regarding apical extrusion were addressed. In endodontic treatment temporary intracanal medication like calcium hydroxide should be completely removed for long term success. For analysis, 60 artificial teeth were prepared, filled with calcium hydroxide, and divided into four groups. The teeth were assigned to needle irrigation, irrigation using a sonic device, PIPS with a lower energy setting (10 mJ, 15 Hz), or PIPS with a higher energy setting (25 mJ/40 Hz). For comparison the weight of each tooth was measured before and after calcium hydroxide incorporation, as well as after removing calcium hydroxide using the four different methods. Regarding safety issues another 24 samples were filled with stained calcium hydroxide and embedded in 0.4% agarose gel. Color changes in the agarose gel due to apical extrusion were digitally analysed using Photoshop. No significant differences were found for calcium hydroxide removal between the two laser groups. Sonic assisted removal and needle irrigation resulted in significant less calcium hydroxide removal than both laser groups, with significantly more calcium hydroxide removal in the ultrasonic group than in the needle irrigation group. For apical extrusion the higher laser (25 mJ/40 Hz) group resulted in significant higher color changes of the periapical gel than all other groups. PIPS with the setting of 10 mJ/15 Hz achieved almost complete removal of calcium hydroxide without increasing apical extrusion of the irrigation solution.

## 1. Introduction

In endodontic treatment elimination of bacteria in the root canal is crucial for the long term success of endodontic therapy [1, 2]. This elimination is achieved by means of mechanical root canal preparation, antibacterial irrigation solutions, and temporary intracanal medication. The most common intracanal medication is calcium hydroxide due to its high antibacterial efficacy [3]. Calcium hydroxide is preferably applied for about one week to achieve the best antibacterial effect [4]. However, for a successful clinical endodontic treatment complete removal of calcium hydroxide is important for the achievement of compact sealing of the root canal system [5].

Currently, removal of intracanal medication is accomplished by different endodontic irrigation protocols. Needle irrigation is the most basic procedure to clean the root canal from calcium hydroxide as well as from remaining debris. However, this procedure lacks efficacy in the removal of bacteria and debris within the numerous ramifications in the root canal system [6]. A better access to these ramifications may be achieved by the activation of irrigation solutions. The introduction of ultrasonic devices has greatly improved the accessibility of irrigation solutions to the root canal system. Another recently introduced method for activation is photon induced photoacoustic streaming (PIPS) by means of an Er:YAG laser [7–9].

Thanks to the high absorption of laser energy in the rinsing solution, photoacoustic pressure waves are produced by emitted laser irradiation [10]. These PIPS induced fluid movements are most likely to result in greater penetration into the ramifications of the root canal system. A tapered and stripped tip is used for the targeted activation with an Er:YAG laser. Avoiding contact with the root canal walls and the activation with a laser tip in the most coronal part of the root canal prevent side effects such as cracks or melting of the dentine.

One possible side effect of every rinsing procedure is apical extrusion of the irrigation solution. The harm caused to periapical tissues depends on the irrigation solution and its concentration [11]. An extruded irrigation solution can cause inflammation [12] and in some cases even necrosis of the tissue, resulting in severe peri- and postoperative pain. Besides, this can compromise the healing of apical periodontitis [13]. Thus, in order to avoid extrusion, pressure levels of irrigation solutions should be below the periapical tissue resistance.

The efficacy of PIPS in removing calcium hydroxide and the safety regarding the periapical tissues have not yet been investigated. Therefore the aim of the present study is to evaluate the efficacy and safety of PIPS in a standardized tooth model.

## 2. Materials and Methods

### 2.1. Sample Preparation

Root canals of 84 acrylic molar teeth were instrumented with ProTaper files (Dentsply, York, PA) to size F3. The weight of the teeth was directly assessed immediately after the preparation. Subsequently, the root canals in 60 teeth were filled with calcium hydroxide (AH Temp, Dentsply, York, PA) and an additional weighting was performed. In 24 teeth the root canals were filled with calcium hydroxide in combination with a red dye (Caries detector, Kuraray, Japan).

### 2.2. Calcium Hydroxide Removal

**Figure 1:**
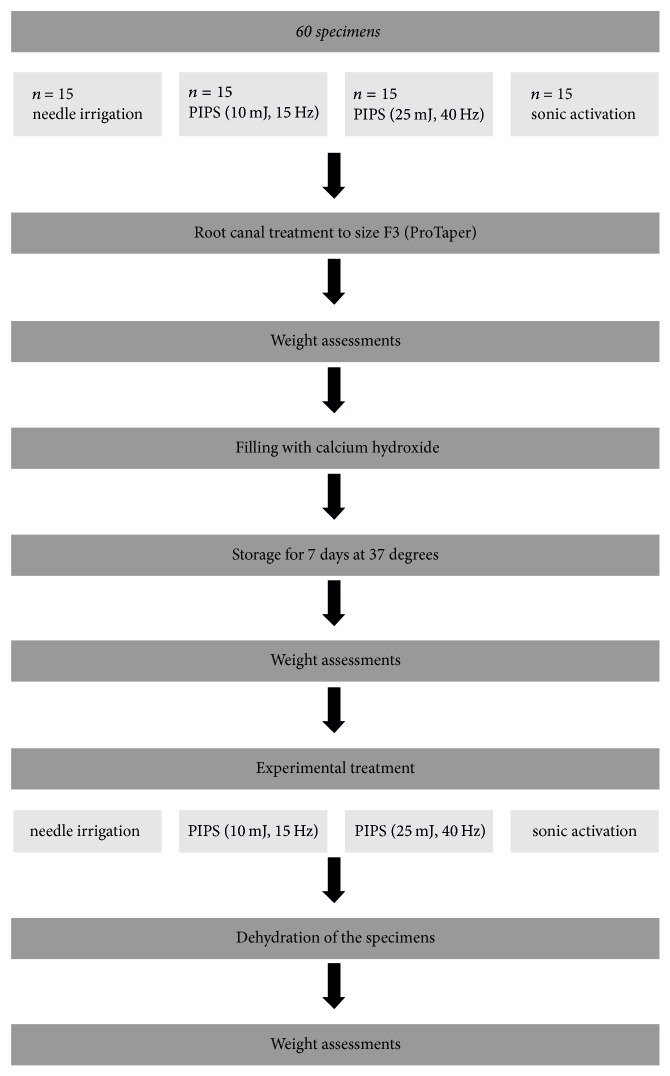
The illustration shows the experimental protocol of the in vitro study.

The 60 teeth filled with calcium hydroxide were divided into four groups.* Group 1*: each root canal was irrigated with 3 ml of sodium hypochlorite (1%) for 1 min. A side vented needle (Perio/Endo Irrigation Needle, KerrHawe, Switzerland) was used for irrigation. The needle was inserted in the canal within 2 mm of working length without binding, and an up and down motion was performed while irrigating.* Group 2* had the same irrigation protocol as group 1 and additionally underwent three times activation of the rinsing solution for 5 seconds with PIPS. An Er:YAG laser with a wavelength of 2940 nm (Lightwalker, Fotona, Ljubljana, Slovenia) was used. The power settings of the Er:YAG laser were 10 mJ, 15 Hz, and 0.15 W with a PIPS tip.* Group 3* had the same irrigation protocol as the other groups and additionally underwent three times laser activation of 5 seconds with the following power settings: 25 mJ, 40 Hz, and 1 W using a PIPS tip.* Group 4* had the same irrigation protocol as the other groups and additionally underwent a sonic activation (Sonicflex, Endo Clean 20, Kavo, Biberach, Germany) for 15 seconds. After the irrigation the teeth were dried and tooth weight was assessed again (Figure 1).

### 2.3. Extrusion Assessment

The 24 teeth filled with calcium hydroxide/dye were embedded in a 0.4% agarose gel (Life Technologies, Waltham, Massachusetts, USA) in transparent plastic tubes. The irrigation protocols for groups 1–4 were performed accordingly. Immediately after the different irrigation procedures the teeth were photographed in a standardized setting from a buccal and a proximal orientation. The images were analysed for extrusion of the dye into the agarose gel using Photoshop software (Adobe, San Jose, USA).

## 3. Results

### 3.1. Calcium Hydroxide Removal

**Figure 2:**
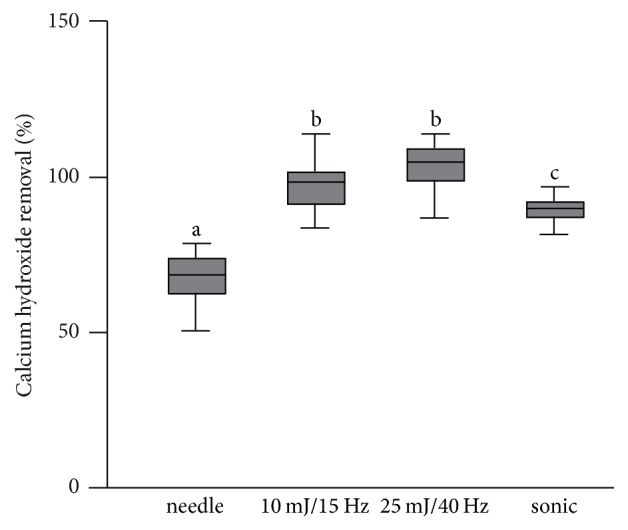
Calcium hydroxide removal of the four groups. Both laser groups resulted in more calcium hydroxide removal than the needle irrigation and the sonic irrigation. Different letters indicate significant differences between the groups.

Using the laser-technique for removing calcium hydroxide resulted in an almost complete removal. In the 10 mJ, 15 Hz PIPS group 99,5% ±10.4% of the calcium hydroxide was removed, which was similar to the 25 mJ, 40 Hz group with a removal rate of 106.7% ±12.4%. No significant differences between the PIPS groups could be detected (*p* > 0.05). In the needle irrigation group 69% ±8.6% of the calcium hydroxide was removed, showing significant differences to both laser groups and the sonic group (all *p* < 0.001). Using sonic activation for removing calcium hydroxide resulted in a removal of 90% ±3,6% which was significantly different to all other groups (*p* < 0.05) (Figure 2).

### 3.2. Extrusion Assessment

**Figure 3:**
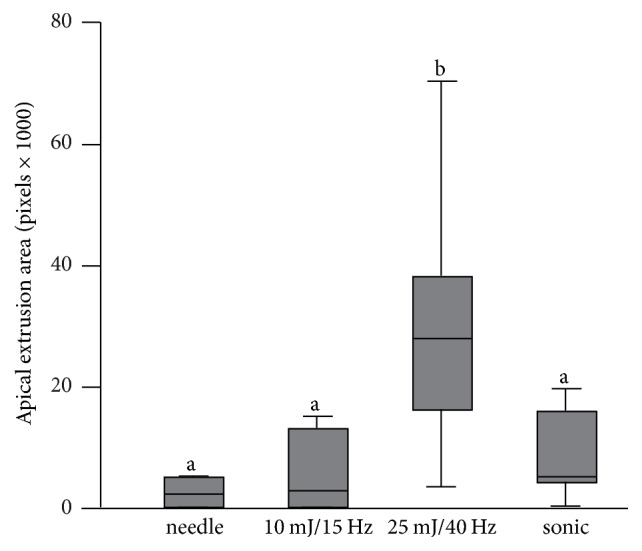
Area of stained irrigation solution due to apical extrusion. The higher laser setting resulted in increased apical extrusion, compared to the lower laser setting, the needle irrigation, and the sonic irrigation. Different letters indicate significant differences between the groups.

**Figure 4:**
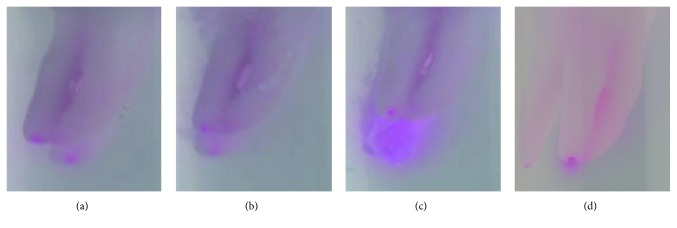
Apical extrusion of colored calcium hydroxide after rinsing with sodium hypochlorite using needle irrigation (a), PIPS 10 mJ/15 Hz (b), PIPS 25 mJ/40 Hz (c), and sonic irrigation (d).

For safety issues extrusion assessment was performed using Photoshop software. In the 10 mJ, 15 Hz PIPS group a colored area of 5722 pixels ± 6977 pixels was seen on the digitized photos. For the 25 mJ, 40 Hz laser group an area of 30660 pixels ± 23303 pixels was stained. There was a significant difference with a *p* value of 0.02 between the two laser groups, showing less extrusion in the 10 mJ, 15 Hz group. The needle irrigation group resulted in a stained area of 4232 pixels ± 5706 pixels, which was significantly lower than the 25 mJ, 40 Hz laser group (*p* < 0.05). However, no significant difference was found between the needle irrigation group and the 10 mJ, 15 Hz laser group. The sonic irrigation group showed a colored area of 8281 pixels ± 7531 pixels resulting in significantly fewer pixels than the 25 mJ, 40 Hz laser group (*p* < 0.05) (Figures 3 and 4).

## 4. Discussion

Success of endodontic treatment depends, inter alia, not only on intracanal medication but also on the removal of these intracanal dressings. Our study compared the capability of an Er:YAG laser device using PIPS with two different power settings with that of needle irrigation and of sonic irrigation in the removal of calcium hydroxide from the root canal. The results showed that both laser settings improved the removal of calcium hydroxide as compared to both, the needle and the sonic irrigation. Furthermore, it could be shown that the lower laser setting was as good as the higher power setting. However, with regard to safety issues, the lower laser setting resulted in significantly less apical extrusion of the rinsing solution.

Our results showing less removal of calcium hydroxide with needle irrigation than with sonic activation or the laser are consistent with recent literature. Several studies have demonstrated that calcium hydroxide was less effectively removed with needle irrigation in comparison to other means as passive ultrasonic irrigation, EndoActivator or RinsEndo [14–16]. PIPS with two different power settings resulted in an almost complete removal of calcium hydroxide. Similarly, Li et al. [17] reported removal of calcium hydroxide for PIPS and needle irrigation in 99 and 81 percent, respectively. This is consistent with our results, which showed an almost 100 percent removal of calcium hydroxide in the PIPS groups, a 90 percent removal in the sonic group, and a 70 percent removal in the needle irrigation group. What is a little bit confusing is the fact that in the higher power setting teeth even weight less than before the application of calcium hydroxide. One explanation might be that an additional removal of debris from the root canal preparation happened. Furthermore it could be that the acrylic tooth itself was altered by the laser irrigation. However this is very unlikely since the laser tip is placed in the irrigation solution during the whole procedure and never contacted the acrylic tooth. Furthermore, no signs of melting or destruction were seen and the whole root canal system was completely filled with irrigation solution during the activation of the laser.

Regarding our results on safety the extrusion of sodium hypochlorite after needle and sonic irrigation was in line with the existing literature [18]. In our model almost no color was detectable, which indicates that no sodium hypochlorite was extruded to the surrounding areas. Yost et al. [19] also found very low extrusion values for needle irrigation. On the other hand, in their study, the use of PIPS with two different power settings (0.15 W, 1 W) resulted in a tenfold increase of extruded rinsing solution. However, results in our experimental design only showed significantly increased extrusion values with the higher power setting of 1 W. When using the lower power setting of 0.15 W no difference to the needle irrigation was observed.

The evaluation of the presence of calcium hydroxide was performed by weighing. The weight of the artificial teeth was measured both before and after loading the calcium hydroxide and after the irrigation procedures. Therefore it was possible to obtain very accurate measurements for the statistical analysis. Radiographic assessment or microscopic imaging techniques are other methods described in the literature for measuring removal efficacy for calcium hydroxide or dentinal debris [20, 21]. Weighing the remaining material allowed us to quantify the results in a very precise way.

A limitation of the present study could be the in vitro design with the use of artificial teeth. But on the other hand this also represents an advantage, since it allows for improved standardization possibilities. Future research should also include extracted human teeth or in vivo models. Also a comparison of PIPS with further activation systems regarding both removal and safety issues can be of interest. Furthermore, considering differences in the absorption of laser energy, the use of different rinsing solutions like chlorhexidine or EDTA might result in different outcomes.

## 5. Conclusion

Within the limitations of the present in vitro study, it can be concluded that PIPS with 0.15 W and 1 W constitutes a very effective removal of calcium hydroxide from the root canal of a standardized tooth model (nearly 100%). Regarding the apical extrusion of irrigation solution the 0.15 W power setting was as safe as needle and sonic irrigation. However the apical extrusion of the irrigation solution was significantly higher in the 1 W laser energy group.
